# Social Well-Being and Quality of Life Among Older Adults in Latvia—A Country with the Lowest Healthy Life Years in the EU

**DOI:** 10.3390/medicina62040634

**Published:** 2026-03-26

**Authors:** Laura Maļina, Anda Ķīvīte-Urtāne, Aija Bukova-Žideļūna

**Affiliations:** Institute of Public Health, Rīga Stradiņš University, Kronvalda Boulevard 9, LV-1010 Rīga, Latvia; anda.kivite-urtane@rsu.lv (A.Ķ.-U.); aija.bukova@rsu.lv (A.B.-Ž.)

**Keywords:** quality of life, CASP-12, social well-being, older adults, SHARE study

## Abstract

*Background and Objectives*: Population ageing is a major challenge of the 21st century and is associated with declining physical and mental abilities, increased disease burden, and higher mortality. Latvia has the lowest healthy life expectancy in the European Union. Social well-being is an important component of healthy and active ageing and may be associated with older adults’ quality of life (QoL). This study aimed to assess the relationship between social well-being, as a component of health, and QoL, including its components (control, autonomy, self-realisation and pleasure), among adults aged 50 and older in Latvia. *Materials and Methods*: Data from 1643 Latvian participants in wave 9 of the Survey of Health, Ageing, and Retirement in Europe (2022) were analysed using linear regression. QoL was measured using the 12-item Control, Autonomy, Self-Realisation, and Pleasure (CASP-12) scale. Social well-being factors included household composition, education, employment status, financial capacity, living area, social network (SN) characteristics, and received help, based on self-reported questionnaires. Results were considered statistically significant if the *p*-value was less than 0.05. *Results*: The factors positively associated with overall QoL were being employed, better financial capacity, greater satisfaction with SN, larger SN, participation in social activities, and higher educational attainment. Being employed and the ability to make ends meet easily were positively associated with all QoL components. Higher satisfaction with the SN and participation in social activities were positively related to the control, autonomy, pleasure, and self-realisation components. *Conclusions*: These findings underscore the importance of social and economic resources for QoL in later adulthood, suggesting that both the quality of social relationships and material security play a central role in shaping overall QoL and its components among older adults.

## 1. Introduction

Population ageing is one of the major challenges of the 21st century. The proportion of people aged 65 and older is increasing faster than the number of people under 65, creating growing demands on health and social systems [1,2]. The population aged 65 years and over in the European Union (EU) increased by an average of 2.9% between 2014 and 2024, whereas in Latvia, this increase was 2.2% [3]. However, in 2023, the average healthy life years at birth in the EU was 63.3 years for women and 62.8 years for men, while in Latvia it was 54.3 years for women and 51.2 years for men, making it the lowest in the EU [4]. These demographic and health trends highlight the importance of understanding the factors that shape quality of life (QoL) in later adulthood.

Health is a state of complete physical, mental and social well-being, not merely the absence of disease or infirmity [5] and is an important component of healthy and active ageing [6]. The World Health Organization defines healthy ageing as the process of developing and maintaining functional ability that enables well-being in older age [7]. Within this framework, QoL reflects individuals’ perceptions of their position in life in relation to their goals, expectations, and social context [8]. Among older adults, QoL is often conceptualised through dimensions such as control, autonomy, self-realisation, and pleasure, which reflect the ability to maintain independence and experience well-being in daily life [9]. Previous research has shown that QoL in older adults is associated with physical and mental health, including chronic conditions, functional limitations, and mental health problems [10,11,12,13,14,15,16], while the social dimension of well-being has been examined less frequently.

As a multidisciplinary concept, social well-being encompasses aspects such as social relationships, social participation, and social support, which may influence well-being in later life [17]. These dimensions are often operationalised through indicators reflecting both the structural and qualitative aspects of social relationships. Characteristics of the social network (SN), social support, participation in social activities, and demographic factors may play important roles in the QoL of older people. For example, people living alone [18] or without a partner in the household [19] often report lower QoL. Therefore, it is important to be socially active in old age. Participating in at least one social activity, such as voluntary work, sports activities or political organisations, is associated with a higher QoL [20]. Participation in social activities can increase SN size. Research shows that a larger SN is associated with higher QoL and greater satisfaction with it [19,20,21]. Individuals with larger SN sizes tend to receive social and emotional support more frequently [22,23]. However, studies have identified a negative correlation between the availability of social support and QoL [20], which can be explained by other health concerns like physical decline and mental health illnesses [23].

The QoL of older adults may also be associated with demographic and socio-economic status (SES) factors. Studies show that men usually report better QoL than women [20,24], that older age is a significant factor in lower QoL [19,24], and that higher educational level is associated with a higher QoL [19,20,24]. Unemployed older adults and those experiencing financial problems have lower levels of QoL [20].

The scientific literature identifies factors associated with social well-being and QoL among older individuals; however, there is limited evidence on the associations between social well-being and components of QoL. This study aimed to assess how social well-being, as a component of health, is associated with overall QoL and its components (control, autonomy, self-realisation and pleasure) among adults aged 50 years and older in Latvia, a country with the lowest healthy life expectancy in the EU.

## 2. Materials and Methods

### 2.1. Study Design and Sample

Data from the Survey of Health, Ageing, and Retirement in Europe (SHARE) were used in this study [25]. SHARE is a multidisciplinary, cross-national longitudinal panel study that collects data about determinants of population ageing, covering areas such as health, economics, demography, social and family networks, social support, and other [26]. The data analysis of this study uses cross-sectional data from wave 9 of SHARE [25], with data collected using computer-assisted personal interviews (CAPI) with respondents in their households, conducted in 2022 [27,28].

The Latvian SHARE wave 9 sample included 1716 respondents. Adults aged 50 years and older who completed all items of the QoL scale were included in the analysis; thus, the study sample comprised 1643 respondents from Latvia.

### 2.2. Variables

The main outcome variable, QoL in older adults, was measured using the 12-item Control, Autonomy, Self-Realisation, and Pleasure (CASP-12) scale. This scale is a modification of the original CASP-19 [9,29]. The CASP scale has been used in various ageing studies [30,31], ensuring the possibility of data comparison. The CASP-12 is a 12-item scale, with each item assessed on a four-point Likert scale, comprising four components (control, autonomy, self-realisation and pleasure) [32]. Components of control and autonomy are associated with feelings arising from active participation in society. In contrast, self-realisation and pleasure represent the extent to which these feelings of freedom can be actualised [29]. The four subscales of components range from 4 to 12 points, and the overall CASP-12 from 12 to 48 points, with higher scores indicating better QoL [32].

Demographic and SES variables included gender (woman vs. man), age group (50–64 years; 65–74 years; 75+ years) (with 75+ years used as the reference category in regression analysis) [19], household composition (living with someone else vs. living alone) [24], employment status (employed vs. not employed), and living area (urban vs. rural). Educational level was classified according to the International Standard Classification of Educational Degrees (ISCED-97) [33] and categorised into three groups: low (ISCED 1–2), medium (ISCED 3–4), and high (ISCED 5–6), with low education used as the reference category. The financial capacity was derived from respondents’ reported ability to make ends meet and categorised as easily vs. with difficulty.

Social well-being indicators included in the analysis were SN characteristics, received help and participation in social activities. Variables describing SN, including network size, satisfaction with the SN, and emotional closeness to the closest SN member, were used as indicators of social relationships and social integration. The size of the SN was measured as the number of individuals with whom respondents had the most frequent contact during the last 12 months. Respondents could report up to seven individuals, resulting in an SN size ranging from 0 to 7. Satisfaction with the SN was derived from a question assessing respondents’ satisfaction with their relationships with SN members. In the SHARE questionnaire, this variable is assessed using a 10-point Likert scale, with higher scores indicating greater satisfaction [34]. In the present study, to account for the skewed distribution and ceiling effect, the variable was categorised into three groups: low/medium (0–7 points), high (8–9 points), and very high (10 points), with low/medium satisfaction used as the reference category. The respondents were asked to evaluate the closeness of their relationship with the closest member of their SN. The response options were categorised into two groups (higher closeness vs. lower closeness).

Help received was derived from two questions that asked whether the respondent had received any help from someone living within or outside the household during the past year. If the respondent answered positively to either question, the respective variable was coded as a two-category variable (yes vs. no).

The number of social activities was calculated as the sum of scores for five activities performed in the past year, each of which required leaving the house or interacting with other people. These activities were: 1. “Done voluntary or charity work”, 2. “Attended an educational or training course”, 3. “Gone to a sport, social or other kind of club”, 4. “Taken part in a political or community-related organisation”, 5. “Played cards or games such as chess”. A score ranging from 0 to 5 was calculated for the total number of social activities conducted in the last 12 months [35]. To account for the skewed distribution and strong floor effect, this variable was categorised into two groups: participation (one or more activities) vs. no participation (no activities).

### 2.3. Statistical Analysis

Descriptive statistics were used to describe the study sample. For categorical variables, percentage distributions were determined, and 95% confidence intervals (95% CI) were calculated. For continuous variables, data normality was assessed using the Shapiro–Wilk test, and results were summarised as medians with interquartile ranges (IQR).

Initially, univariate linear regression analyses were conducted to examine the associations between each independent variable and each outcome variable. Regression diagnostics were performed to assess the assumptions of the linear models, including inspection of residual plots. Separate regression models were estimated for the overall QoL score and for each of its components (control, autonomy, pleasure, and self-realisation). Prior to multivariate regression, collinearity diagnostics were conducted. Variance inflation factors (VIF) and tolerance values were used to assess collinearity among independent variables. No collinearity was observed, indicating that all variables could be included in the model.

All independent variables were included in the multivariate regression models. Multivariate regression analyses were conducted using listwise deletion, meaning that cases with missing values for any variable included in the model were excluded. The final analytical sample for the multivariate linear regression analyses consisted of 1515 respondents. *p*-values were reported for both univariate and multivariate analyses to provide a comprehensive overview of the relationships between variables.

IBM SPSS Statistics Version 29.0.0.0 was used for data analysis. The analyses were conducted using weighted data. Cross-sectional weights provided by SHARE were applied to account for sampling design and to adjust the sample to the population structure based on age group, gender, and region [34]. The 95% CI of proportions were calculated using the Epitools 95% proportion calculator [36]. Results were considered statistically significant if the *p*-value was less than 0.05.

## 3. Results

### 3.1. Characteristics of the Total Sample

The total sample of the study (Table 1) included 1643 individuals aged 50 years and older from Latvia. Of these, 61.29% were women, 40.23% were aged between 50 and 64 years, and 70.72% lived with at least one other household member. A total of 70.75% of respondents were not employed. Regarding financial capacity, 61.23% of respondents acknowledged experiencing making ends meet with difficulty. In terms of SN characteristics, the median SN size was 1 person, and 42.75% respondents reported high satisfaction (8–9 points) with their SN. In total, 87.40% had not received any help from someone living within or outside the household during the past year. Although the maximum possible number of social activities performed last year could have been 5, among Latvian respondents, it ranged from 0 to 4. Furthermore, 81.04% of respondents reported that they had not participated in any social activities during the past year (Table 1).

The median QoL score across the study sample was 34 points, with scores ranging from 16 to 48. Of the QoL components, pleasure had the highest median score (10 points), followed by self-realisation (9 points). The median score for both control and autonomy were 8 points (Table 1).

### 3.2. QoL and Associated Factors

Of all the factors analysed in the multivariate analyses (Table 2), the three factors with the highest regression coefficients positively associated with QoL were being employed (vs. not employed) (β^a^ = 0.23, *p* < 0.001); very high satisfaction with SN (vs. low/medium satisfaction) (β^a^ = 0.28, *p* < 0.001); and higher educational level (vs. low) (β^a^ = 0.23, *p* < 0.001).

The factors with the highest regression coefficients positively associated with the control component were very high satisfaction with SN (vs. low/medium satisfaction) (β^a^ = 0.28, *p* < 0.001); ability to make ends meet easily (vs. with difficulty) (β^a^ = 0.19, *p* < 0.001); and 50–64 age group (vs. 75+ years) (β^a^ = 0.18, *p* < 0.001). An increase in the size of SN by one person was negatively associated with the control component (β^a^ = −0.18, *p* < 0.001).

The main factors that increased the autonomy were the ability to make ends meet easily (vs. with difficulty) (β^a^ = 0.29, *p* < 0.001) and the SN size (increasing by one person) (β^a^ = 0.22, *p* < 0.001). However, living with others in the same household was a negative factor compared with respondents who lived alone (β^a^ = −0.21, *p* < 0.001).

For the component pleasure, the three factors with the highest regression coefficients were size of SN (increasing by one person) (β^a^ = 0.36, *p* < 0.001); higher educational level (vs. low) (β^a^ = 0.21, *p* < 0.001); and satisfaction with SN—high vs. low/medium satisfaction (β^a^ = 0.21, *p* < 0.001) and very high vs. low/medium satisfaction (β^a^ = 0.22, *p* < 0.001).

The three factors with the highest regression coefficients associated with component self-realisation were being employed (vs. not employed) (β^a^ = 0.27, *p* < 0.001); higher educational level (vs. low) (β^a^ = 0.23, *p* < 0.001); and very high satisfaction with SN (vs. low/medium satisfaction) (β^a^ = 0.21, *p* < 0.001). The full regression results for each model are provided in the Supplementary Tables (Tables S1–S5).

## 4. Discussion

The aim of this study was to assess the relationship between social well-being and QoL, including its components, among adults aged 50 and older in Latvia. In this study, QoL was measured using the CASP-12 scale, which ranges from 12 to 48 points. Higher scores indicate better QoL. The median QoL score across the study sample was 34 points, whereas the mean QoL score in countries participating in SHARE was 37.06 points (2015 data) [24]. However, this comparison should be interpreted as contextual rather than inferential, as differences in survey waves, weighting procedures, and age composition across samples may influence these estimates. The four QoL subscale components (control, autonomy, self-realisation, and pleasure) ranged from 4 to 12 points. Median scores were 10 for pleasure, 9 for self-realisation, and 8 for both control and autonomy.

The findings of this study indicate that several SES factors and SN characteristics are associated with QoL among adults aged 50 years and older in Latvia. In particular, having better financial situation, being employed, having higher educational level, higher satisfaction with SN and participation in social activities were positively associated with overall QoL.

### 4.1. Demographic Factors

#### 4.1.1. Gender

In line with previous studies [20,24], this study found that men reported higher QoL than women. When analysing the relationship between gender and individual components of QoL, women reported lower autonomy and self-realisation but higher control. Compared with men, women are more likely to be widowed and to face situations that require independent decision-making [37], which may negatively influence their perceived autonomy and opportunities for self-realisation. Similar findings have been reported in a study across 28 European countries, where men had higher average autonomy scores than women [38]. While men tend to have greater physical independence [39], women’s autonomy may be dependent on social and psychological resources [40].

#### 4.1.2. Age

Results from various studies confirm that as an individual’s age increases, QoL deteriorates [19,24]. Our study found that people aged 65–74 reported better overall QoL than those aged 75 and older, with a slightly stronger association compared to the younger age group (50–64 years). This can be explained by the third age phenomenon, which refers to the period between retirement and the onset of age-related limitations, characterised by relatively good health, sufficient resources, and opportunities for self-fulfilment, autonomy, and meaningful engagement [41].

The characteristics of the third age phenomenon are also evident in the fact that respondents in younger age groups reported higher self-realisation and control scores compared to those aged 75 and older. As individuals age, control declines, which can be attributed to the deterioration of physical and mental health [42,43]. At the same time, individuals in the younger age groups reported lower levels of autonomy and pleasure compared to those aged 75 and older, suggesting that different dimensions of QoL may change in district ways across age groups.

### 4.2. SES Factors

#### 4.2.1. Household Composition

In this study, individuals living alone reported higher overall QoL compared to those living with someone else. At the same time, individuals living alone reported higher autonomy and pleasure but lower control and self-realisation scores than those living with someone else. Higher autonomy may be explained by the fact that living alone allows individuals to feel a sense of self-determination, as fewer external obligations and constraints, for example, fulfilling family responsibilities, exist [44]. Consequently, lower control and self-realisation scores may reflect that older adults living alone often experience social isolation and loneliness, thereby reducing their sense of control over their lives [45].

#### 4.2.2. Educational Level

Educational level was significantly associated with participants’ QoL: a higher educational level was associated with higher QoL, consistent with previous studies [19,20,24]. The conceptual framework for general and health literacy, defined by Rootman and Ronson [46], proposes that education is one of the determinants influencing a range of personal lifestyle choices, which can lead to higher rates of QoL.

Higher education was also associated with higher scores for the control, autonomy, pleasure, and self-realisation components. The results could be explained by the fact that higher levels of education are usually associated with a higher subjective social status and later-life socioeconomic advantage [47]. SES variables included in the models (education, employment status, and financial capacity) may be conceptually related and could partly reflect interconnected pathways influencing QoL in later life.

#### 4.2.3. Employment Status and Financial Capacity

As in the study by Lestari et al. [20], this study shows that employed individuals (vs. not employed) and those who find it easy to make ends meet (vs. those with difficulties) reported better QoL. The sample of this study included individuals in pre-retirement age (50–64 years), for whom employment represents a normative life-course stage rather than an indicator of active ageing. Therefore, the observed association between employment and QoL may partly reflect age composition and socioeconomic differentials. The literature suggests that higher income is a major factor in better QoL [19,24].

Individuals who were being employed and experienced no difficulty in making ends meet also reported higher rates for all QoL components. These associations may be explained by the fact that income stability and reduced economic strain can strengthen perceived control and autonomy. In addition, greater economic resources may provide older adults with more opportunities to maintain independence and remain socially engaged. Social roles can foster purpose, continuity, and social integration in later life [48], thereby supporting pleasure and self-realisation.

#### 4.2.4. Living Area

This study found that respondents living in urban areas reported higher overall QoL than those living in rural areas. However, individuals living in rural areas reported lower scores on the control component but higher scores on autonomy and self-realisation (vs. living in urban areas). People living in rural areas may have limited access to services and infrastructure [49]. This can limit their ability to influence their daily living conditions, thereby negatively affecting their sense of control. Concurrently, rural environments can promote greater autonomy and self-realisation through closer social ties, a stronger sense of belonging, and opportunities to engage in meaningful, identity-building activities that provide a deeper sense of purpose in life [23].

### 4.3. Characteristic of SN

#### 4.3.1. SN Size, Satisfaction, and Emotional Closeness

SN size and satisfaction with it, as well as higher emotional closeness, were positively associated with respondents’ QoL. These findings highlight the distinction between the size of the SN and the quality of social relationships. While SN size reflects the number of social contacts, satisfaction with SN and emotional closeness capture the qualitative aspects of social ties. The median SN size in the study population was low, with only one person. Results from a similar study indicate that the mean size of SN in countries participating in SHARE was 2.61 people (2015 data) [24]. However, 42.75% respondents reported high (8–9 points) satisfaction with their SN.

According to the socioemotional selectivity theory [50], as people age, SN size gradually declines, primarily by excluding acquaintances from their social circle. This phenomenon can be influenced by a limited time perspective and by geographical movement, for example. Concurrently, closer relationships, such as those with children, spouses, and other family members, mainly remain unaltered or evolve towards greater intimacy over time. Additionally, previous studies have found that larger SN size and satisfaction with it are associated with higher QoL [19,20,21], greater emotional closeness to SN members [24]. The SN measure used in SHARE allows respondents to report up to seven SN members and therefore reflects the most salient social ties rather than the full breadth of an individual’s SN, which may reduce variability and potentially diminish the strength of observed associations.

An increase in the size of the SN decreases control but increases autonomy, pleasure, and self-realisation. This pattern may reflect the multidimensional nature of QoL, where structural and qualitative aspects of social relationships may be associated with different components of QoL in distinct ways. Conversely, higher satisfaction with the SN is associated with higher levels across all QoL components, whereas greater emotional closeness is associated with higher autonomy, pleasure, and self-realisation, but lower control. A larger SN may create more social obligations, which can reduce subjective control over daily decisions, even if the support provided by the SN contributes to reduced loneliness [51], which could explain higher levels of pleasure. At the same time, an increase in autonomy and self-realisation can be explained by the resources and support provided by SN [23], which enable individuals to achieve their goals and make their own choices.

#### 4.3.2. Help Received

This study reports that 12.6% of respondents reported receiving some form of help (e.g., personal care, practical household assistance, help with paperwork) in the past year, whereas another study reports a rate of 21.3% (2017 data from 16 countries) [20]. Despite differences in the proportions of help received, our results and those of Lestari et al. [20] were similar, indicating a negative association between receiving help and QoL. The observed negative association between receiving help and QoL should be interpreted with caution. Lower QoL among individuals who received help may reflect underlying functional limitations or poorer health status [10,11,12,13,14,15,16], rather than the effect of receiving assistance itself, particularly as the present models did not include direct indicators of physical or mental health.

Older adults who had received help reported lower control, pleasure, and self-realisation scores. These associations should be interpreted with caution, as it may reflect underlying functional limitations or poorer health status rather than the effect of receiving assistance itself. While receiving support may be perceived as a loss of independence in some cases [52], the observed relationship in this study is likely associated with unmeasured health-related factors.

#### 4.3.3. Social Activities

Study participants could have performed a maximum of five social activities in the past year: voluntary or charity work; an educational or training course; participation in a sports or social club; involvement in a political or community organisation; playing cards or games. However, among Latvian respondents, the number of activities ranged from 0 to 4. A total of 81.04% of respondents reported that they had not participated in any social activities during the past year.

Our study indicates that participation in at least one social activity (vs. none) was positively associated with QoL, which is consistent with previous studies [20,53] and the activity theory of ageing [54]. This theory explains that keeping older people socially active delays the ageing process and improves their QoL.

Participation in at least one social activity (vs. none) was also associated with higher scores for the control, autonomy, pleasure, and self-realisation components. These results can be explained by self-determination theory [55]. This theory emphasises the importance of autonomy, competence, and social connection in promoting well-being. Social participation in older age contributes to the satisfaction of these needs by strengthening the subjective sense of control and autonomy, providing opportunities for self-realisation, and promoting positive emotional experiences.

### 4.4. Strengths and Limitations

The present study has a few limitations that should be noted. First, the cross-sectional design prevented us from drawing conclusions about causality. Given that SHARE is a large, cross-country European study using a standardised questionnaire, it would be valuable to analyse the data longitudinally and across other countries participating in SHARE. Secondly, the SHARE questionnaire is based on self-reported data, which may have introduced bias into the results. Thirdly, other covariates, including physical and mental health measures, may have influenced the relationship between the independent variables and QoL, including its components.

A major strength of this study is that the association between social well-being and QoL was analysed not only at a general level, but also in relation to components of QoL—control, autonomy, pleasure, and self-realisation. Furthermore, the linkage of individual-level data and the consideration of a wide range of factors strengthen the robustness and depth of the findings.

## 5. Conclusions

The aim of this study was to examine how social well-being is associated with overall QoL and its components (control, autonomy, self-realisation, and pleasure) among adults aged 50 years and older in Latvia. The results indicate that being employed, better financial capacity, greater satisfaction with SN, larger SN, participation in social activities, and higher educational level were associated with higher overall QoL, highlighting the importance of both economic resources and social well-being in later adulthood.

The analysis of individual QoL components showed that different aspects of social well-being are related to specific dimensions of QoL. Qualitative aspects of social relationships, such as satisfaction with SN and emotional closeness, were positively associated with several QoL components, suggesting that the quality of social ties may play a particularly important role in shaping QoL in later life.

These findings highlight the importance of policies and programmes that encourage social participation, support meaningful interpersonal relationships, and promote community integration among older adults. While the present study focused on social well-being and QoL, future research may extend this approach by integrating physical and mental health dimensions to provide a more comprehensive understanding of QoL in older age.

## Figures and Tables

**Table 1:** Characteristics of the study sample, Latvia (n = 1643).

	% (n)	95% CI	Missing % (n)
**Gender**			
Man	38.71 (636)	36.38–41.09	0 (0)
Woman	61.29 (1007)	58.91–63.62	
**Age group**			
50–64 years	40.23 (661)	37.89–42.62	0 (0)
65–74 years	33.05 (543)	30.82–35.36	
75+ years	26.72 (439)	24.64–28.91	
**Household composition**			
Living with someone else	70.72 (1162)	68.48–72.87	0 (0)
Living alone	29.28 (481)	27.13–31.52	
Educational level *			
Low	12.76 (208)	11.23–14.47	0.79 (13)
Medium	62.21 (1014)	59.83–64.53	
High	25.03 (408)	22.99–27.19	
Employment status *			
Employed	29.25 (477)	27.09–31.50	0.73 (12)
Not employed	70.75 (1154)	68.50–72.91	
Making ends meet *			
Easily	38.77 (625)	36.42–41.17	1.89 (31)
With difficulty	61.23 (987)	58.83–63.58	
Living area *			
Rural	42.23 (685)	39.85–44.65	1.28 (21)
Urban	57.77 (937)	55.35–60.15	
**Size of SN (0–7 persons), median (IQR)**	1 (1; 3)		0 (0)
**Satisfaction with SN**			
Low/medium (0–7 points)	21.92 (360)	19.99–23.99	0.06 (1)
High (8–9 points)	42.75 (702)	40.38–45.16	
Very high (10 points)	35.32 (580)	33.05–37.67	
Emotional closeness of the closest SN member *			
Lower closeness	40.09 (635)	37.70–42.52	3.60 (59)
Higher closeness	59.91 (949)	57.48–62.30	
**Help received**			
No	87.40 (1436)	85.71–88.92	0 (0)
Yes	12.60 (207)	11.08–14.29	
**Participating in social activities**			
No participation	81.04 (1325)	79.07–82.87	0.49 (8)
Participation	18.96 (310)	17.13–20.93	
**QoL (12–48 points), median (IQR)**	34 (31; 39)		0 (0)
Control (3–12 points), median (IQR)	8 (7; 10)		
Autonomy (3–12 points), median (IQR)	8 (7; 9)		
Pleasure (3–12 points), median (IQR)	10 (9; 12)		
Self-Realisation (3–12 points), median (IQR)	9 (7; 10)		

95% CI, confidence interval; SN, social network; IQR, interquartile range; QoL, quality of life. * The sum of some variables may differ from the total sample size due to missing data.

**Table 2:** Factors related to quality of life and its components in Latvia.

	Control		Autonomy		Pleasure		Self-Realisation		QoL	
	β ^a^	*p*-Value	β ^a^	*p*-Value	β ^a^	*p*-Value	β ^a^	*p*-Value	β ^a^	*p*-Value
**Gender**										
Woman vs. Man	0.01	<0.001	−0.08	<0.001	0.00	0.524	−0.05	<0.001	−0.04	<0.001
**Age group**										
50–64 years vs. 75+ years	0.18	<0.001	−0.09	<0.001	−0.11	<0.001	0.08	<0.001	0.04	<0.001
65–74 years vs. 75+ years	0.09	<0.001	−0.03	<0.001	−0.05	<0.001	0.09	<0.001	0.05	<0.001
**Household composition**										
Living with someone else vs. Living alone	0.11	<0.001	−0.21	<0.001	−0.02	<0.001	0.02	<0.001	−0.02	<0.001
**Educational level**										
Medium vs. Low	0.11	<0.001	0.09	<0.001	0.17	<0.001	0.19	<0.001	0.20	<0.001
High vs. Low	0.09	<0.001	0.12	<0.001	0.21	<0.001	0.23	<0.001	0.23	<0.001
**Employment status**										
Employed vs. Not employed	0.15	<0.001	0.07	<0.001	0.15	<0.001	0.27	<0.001	0.23	<0.001
**Making ends meet**										
Easily vs. With difficulty	0.19	<0.001	0.29	<0.001	0.04	<0.001	0.03	<0.001	0.18	<0.001
**Living area**										
Urban vs. Rural	0.10	<0.001	0.03	<0.001	0.00	0.012	−0.08	<0.001	0.01	<0.001
**Size of SN**										
Increasing by one person	−0.18	<0.001	0.22	<0.001	0.36	<0.001	0.17	<0.001	0.19	<0.001
**Satisfaction with SN**										
High vs. Low/medium	0.08	<0.001	0.06	<0.001	0.21	<0.001	0.17	<0.001	0.19	<0.001
Very high vs. Low/medium	0.28	<0.001	0.07	<0.001	0.22	<0.001	0.21	<0.001	0.28	<0.001
**Emotional closeness of the closest SN member**										
Higher closeness vs. Lower closeness	−0.03	<0.001	0.11	<0.001	0.14	<0.001	0.07	<0.001	0.10	<0.001
**Help received**										
Yes vs. No	−0.08	<0.001	0.06	<0.001	−0.04	<0.001	−0.11	<0.001	−0.07	<0.001
**Participating in social activities**										
Participation vs. No participation	0.13	<0.001	0.07	<0.001	0.07	<0.001	0.18	<0.001	0.16	<0.001

QoL, quality of life; SN, social network; β, standardised regression coefficient from multivariate linear regression models. ^a^ Adjusted for gender, age group, household composition, educational level, employment status, making ends meet, living area, size of SN, satisfaction with SN, emotional closeness of the closest SN member, help received, participating in social activities. Model fit (multivariate models): Control (R^2^ = 0.28, adjusted R^2^ = 0.28); Autonomy (R^2^ = 0.28, adjusted R^2^ = 0.28); Pleasure (R^2^ = 0.38, adjusted R^2^ = 0.38); Self-Realisation (R^2^ = 0.36, adjusted R^2^ = 0.36); QoL (R^2^ = 0.43, adjusted R^2^ = 0.43).

## Data Availability

The data used in this study are derived from the Survey of Health, Ageing, and Retirement in Europe (SHARE) and are publicly available. Data can be accessed through the official SHARE website (https://share-eric.eu/data/) upon registration and compliance with the data access requirements.

## Supplementary Materials

The following supporting information can be downloaded at: https://www.mdpi.com/article/10.3390/medicina62040634/s1, Table S1: Factors related to control component in Latvia; Table S2: Factors related to autonomy component in Latvia; Table S3: Factors related to pleasure component in Latvia; Table S4: Factors related to self-realisation component in Latvia; Table S5: Factors related to quality of life in Latvia.

## Abbreviations

The following abbreviations are used in this manuscript:

EU	European Union
QoL	Quality of life
SES	Socio-economic status
SN	Social network
SHARE	Survey of Health, Ageing, and Retirement in Europe
CAPI	Computer-assisted personal interviews
ISCED-97	International Standard Classification of Educational Degrees
95% CI	95% confidence interval
IQR	Interquartile ranges
