# Supplementary material for: Social Well-Being and Quality of Life Among Older Adults in Latvia—A Country with the Lowest Healthy Life Years in the EU
Source: Medicina (Kaunas). 2026 Mar 26;62(4):634. doi: 10.3390/medicina62040634 (PMC13118212; doi:10.3390/medicina62040634)
Supplement: Supplementary file 1 [file medicina-62-00634-s001.zip › medicina-4193493-supplementary.pdf]

SUPPLEMENTARY MATERIAL

a Table S1. Factors related to control component in Latvia
b Table S2. Factors related to autonomy component in Latvia
c Table S3. Factors related to pleasure component in Latvia
d Table S4. Factors related to self-realisation component in Latvia
e Table S5. Factors related to quality of life in Latvia

a Table S1. Factors related to control component in Latvia.

	Control					
	Univariate model				Multivariate model	
	**B**	**SE**	**95% CI**	***p*-Value**	**β [a]**	***p*-Value**
**Gender**						
Woman vs. Man	−0.29	0.01	−0.30–−0.28	<0.001	0.01	<0.001
**Age group**						
50–64 years vs. 75+ years	1.82	0.01	1.81–1.83	<0.001	0.18	<0.001
65–74 years vs. 75+ years	0.94	0.01	0.92–0.95	<0.001	0.09	<0.001
**Household composition**						
Living with someone else vs. Living alone	1.07	0.01	1.06–1.08	<0.001	0.11	<0.001
**Educational level**						
Medium vs. Low	1.40	0.01	1.38–1.41	<0.001	0.11	<0.001
High vs. Low	1.66	0.01	1.65–1.68	<0.001	0.09	<0.001
**Employment status**						
Employed vs. Not employed	1.65	0.01	1.64–1.66	<0.001	0.15	<0.001
**Making ends meet**						
Easily vs. With difficulty	1.12	0.01	1.11–1.13	<0.001	0.19	<0.001
**Living area**						
Urban vs. Rural	0.36	0.01	0.35–0.37	<0.001	0.10	<0.001
**Size of SN**						
Increasing by one person	−0.06	0.00	−0.07–−0.06	<0.001	−0.18	<0.001
**Satisfaction with SN**						
High vs. Low/ medium	0.28	0.01	0.26–0.29	<0.001	0.08	<0.001
Very high vs. Low/ medium	0.94	0.01	0.93–0.96	<0.001	0.28	<0.001
**Emotional closeness of the closest SN member**						
Higher closeness vs. Lower closeness	0.42	0.01	0.41–0.43	<0.001	−0.03	<0.001
**Help received**						
Yes vs. No	−1.43	0.01	−1.45–−1.42	<0.001	−0.08	<0.001
**Participating in social activities**						
Participation vs. No participation	1.16	0.01	1.14–1.17	<0.001	0.13	<0.001

SN, social network; B, unstandardised regression coefficient from univariate linear regression models; SE, standard error; 95% CI, 95% confidence interval; β, standardised regression coefficient from multivariate linear regression models. [a] Adjusted for gender, age group, household composition, educational level, employment status, making ends meet, living area, size of SN, satisfaction with SN, emotional closeness of the closest SN member, help received, participating in social activities. Model fit (multivariate models): Control (R^2 = 0.28, adjusted R^2 = 0.28).

b Table S2. Factors related to autonomy component in Latvia.

	Autonomy					
	Univariate model				Multivariate model	
	B	SE	95% CI	*p*-Value	β [a]	*p*-Value
**Gender**						
Woman vs. Man	0.00	0.00	−0.01–0.01	0.758	−0.08	<0.001
**Age group**						
50–64 years vs. 75+ years	−0.25	0.01	−0.26–−0.24	<0.001	−0.09	<0.001
65–74 years vs. 75+ years	−0.09	0.01	−0.10–−0.08	<0.001	−0.03	<0.001
**Household composition**						
Living with someone else vs. Living alone	−0.54	0.00	−0.55–−0.53	<0.001	−0.21	<0.001
**Educational level**						
Medium vs. Low	0.35	0.01	0.34–0.36	<0.001	0.09	<0.001
High vs. Low	0.87	0.01	0.85–0.88	<0.001	0.12	<0.001
**Employment status**						
Employed vs. Not employed	0.20	0.00	0.19–0.21	<0.001	0.07	<0.001
**Making ends meet**						
Easily vs. With difficulty	1.14	0.00	1.14–1.15	<0.001	0.29	<0.001
**Living area**						
Urban vs. Rural	0.35	0.00	0.34–0.36	<0.001	0.03	<0.001
**Size of SN**						
Increasing by one person	0.46	0.00	0.46–0.46	<0.001	0.22	<0.001
**Satisfaction with SN**						
High vs. Low/ medium	0.51	0.01	0.51–0.52	<0.001	0.06	<0.001
Very high vs. Low/ medium	1.00	0.01	0.99–1.01	<0.001	0.07	<0.001
**Emotional closeness of the closest SN member**						
Higher closeness vs. Lower closeness	0.85	0.00	0.84–0.85	<0.001	0.11	<0.001
**Help received**						
Yes vs. No	0.39	0.01	0.38–0.40	<0.001	0.06	<0.001
**Participating in social activities**						
Participation vs. No participation	0.62	0.01	0.61–0.63	<0.001	0.07	<0.001

SN, social network; B, unstandardised regression coefficient from univariate linear regression models; SE, standard error; 95% CI, 95% confidence interval; β, standardised regression coefficient from multivariate linear regression models. [a] Adjusted for gender, age group, household composition, educational level, employment status, making ends meet, living area, size of SN, satisfaction with SN, emotional closeness of the closest SN member, help received, participating in social activities. Model fit (multivariate models): Autonomy ($R^2 = 0.28$, adjusted $R^2 = 0.28$).

c Table S3. Factors related to pleasure component in Latvia.

	Pleasure					
	Univariate model				Multivariate model	
	**B**	**SE**	**95% CI**	***p*-Value**	**β [a]**	***p*-Value**
**Gender**						
Woman vs. Man	0.21	0.01	0.21–0.22	<0.001	0.00	0.524
**Age group**						
50–64 years vs. 75+ years	0.16	0.01	0.15–0.17	<0.001	−0.11	<0.001
65–74 years vs. 75+ years	−0.13	0.01	−0.14–−0.12	<0.001	−0.05	<0.001
**Household composition**						
Living with someone else vs. Living alone	0.37	0.01	0.36–0.38	<0.001	−0.02	<0.001
**Educational level**						
Medium vs. Low	0.97	0.01	0.96–0.98	<0.001	0.17	<0.001
High vs. Low	1.70	0.01	1.68–1.71	<0.001	0.21	<0.001
**Employment status**						
Employed vs. Not employed	0.72	0.01	0.71–0.73	<0.001	0.15	<0.001
**Making ends meet**						
Easily vs. With difficulty	0.64	0.01	0.63–0.65	<0.001	0.04	<0.001
**Living area**						
Urban vs. Rural	0.52	0.01	0.51–0.53	<0.001	0.00	0.012
**Size of SN**						
Increasing by one person	0.92	0.00	0.92–0.92	<0.001	0.36	<0.001
**Satisfaction with SN**						
High vs. Low/ medium	1.53	0.01	1.52–1.54	<0.001	0.21	<0.001
Very high vs. Low/ medium	2.30	0.01	2.27–2.29	<0.001	0.22	<0.001
**Emotional closeness of the closest SN member**						
Higher closeness vs. Lower closeness	1.57	0.00	1.57–1.58	<0.001	0.14	<0.001
**Help received**						
Yes vs. No	−0.30	0.01	−0.31–−0.28	<0.001	−0.04	<0.001
**Participating in social activities**						
Participation vs. No participation	0.92	0.01	0.91–0.93	<0.001	0.07	<0.001

SN, social network; B, unstandardised regression coefficient from univariate linear regression models; SE, standard error; 95% CI, 95% confidence interval; β, standardised regression coefficient from multivariate linear regression models. [a] Adjusted for gender, age group, household composition, educational level, employment status, making ends meet, living area, size of SN, satisfaction with SN, emotional closeness of the closest SN member, help received, participating in social activities. Model fit (multivariate models): Pleasure ($R^2 = 0.38$, adjusted $R^2 = 0.38$).

d Table S4. Factors related to self-realisation component in Latvia.

	Self-Realisation					
	Univariate model				Multivariate model	
	B	SE	95% CI	*p*-Value	β [a]	*p*-Value
**Gender**						
Woman vs. Man	−0.29	0.01	−0.30–−0.28	<0.001	−0.05	<0.001
**Age group**						
50–64 years vs. 75+ years	1.78	0.01	1.77–1.79	<0.001	0.08	<0.001
65–74 years vs. 75+ years	0.80	0.01	0.79–0.81	<0.001	0.09	<0.001
**Household composition**						
Living with someone else vs. Living alone	0.99	0.01	0.98–1.00	<0.001	0.02	<0.001
**Educational level**						
Medium vs. Low	1.61	0.01	1.59–1.62	<0.001	0.19	<0.001
High vs. Low	2.27	0.01	2.25–2.29	<0.001	0.23	<0.001
**Employment status**						
Employed vs. Not employed	1.91	0.01	1.90–1.92	<0.001	0.27	<0.001
**Making ends meet**						
Easily vs. With difficulty	0.79	0.01	0.78–0.80	<0.001	0.03	<0.001
**Living area**						
Urban vs. Rural	−0.02	0.01	−0.03–−0.01	<0.001	−0.08	<0.001
**Size of SN**						
Increasing by one person	0.60	0.00	0.59–0.60	<0.001	0.17	<0.001
**Satisfaction with SN**						
High vs. Low/ medium	1.21	0.01	1.20–1.22	<0.001	0.17	<0.001
Very high vs. Low/ medium	1.85	0.01	1.84–1.86	<0.001	0.21	<0.001
**Emotional closeness of the closest SN member**						
Higher closeness vs. Lower closeness	1.22	0.01	1.21–1.23	<0.001	0.07	<0.001
**Help received**						
Yes vs. No	−1.36	0.01	−1.38–−1.35	<0.001	−0.11	<0.001
**Participating in social activities**						
Participation vs. No participation	1.70	0.01	1.69–1.71	<0.001	0.18	<0.001

SN, social network; B, unstandardised regression coefficient from univariate linear regression models; SE, standard error; 95% CI, 95% confidence interval; β, standardised regression coefficient from multivariate linear regression models. [a] Adjusted for gender, age group, household composition, educational level, employment status, making ends meet, living area, size of SN, satisfaction with SN, emotional closeness of the closest SN member, help received, participating in social activities. Model fit (multivariate models): Self-Realisation ($R^2 = 0.36$, adjusted $R^2 = 0.36$).

e Table S5. Factors related to quality of life in Latvia.

	QoL					
	Univariate model				Multivariate model	
	B	SE	95% CI	*p*-Value	β [a]	*p*-Value
**Gender**						
Woman vs. Man	−0.36	0.01	−0.39–−0.33	<0.001	−0.04	<0.001
**Age group**						
50–64 years vs. 75+ years	3.51	0.02	3.48–3.54	<0.001	0.04	<0.001
65–74 years vs. 75+ years	1.52	0.02	1.48–1.55	<0.001	0.05	<0.001
**Household composition**						
Living with someone else vs. Living alone	1.89	0.01	1.86–1.92	<0.001	-0.02	<0.001
**Educational level**						
Medium vs. Low	4.33	0.02	4.29–4.36	<0.001	0.20	<0.001
High vs. Low	6.50	0.02	6.45–6.53	<0.001	0.23	<0.001
**Employment status**						
Employed vs. Not employed	4.48	0.01	4.46–4.51	<0.001	0.23	<0.001
**Making ends meet**						
Easily vs. With difficulty	3.69	0.01	3.67–3.72	<0.001	0.18	<0.001
**Living area**						
Urban vs. Rural	1.21	0.01	1.18–1.23	<0.001	0.01	<0.001
**Size of SN**						
Increasing by one person	1.91	0.01	1.90–1.92	<0.001	0.19	<0.001
**Satisfaction with SN**						
High vs. Low/ medium	3.53	0.02	3.50–3.56	<0.001	0.19	<0.001
Very high vs. Low/ medium	6.07	0.02	6.04–6.10	<0.001	0.28	<0.001
**Emotional closeness of the closest SN member**						
Higher closeness vs. Lower closeness	4.06	0.01	4.04–4.09	<0.001	0.10	<0.001
**Help received**						
Yes vs. No	−2.71	0.02	−2.74–−2.67	<0.001	−0.07	<0.001
**Participating in social activities**						
Participation vs. No participation	4.39	0.02	4.36–4.42	<0.001	0.16	<0.001

QoL, quality of life; SN, social network; B, unstandardised regression coefficient from univariate linear regression models; SE, standard error; 95% CI, 95% confidence interval; β, standardised regression coefficient from multivariate linear regression models. [a] Adjusted for gender, age group, household composition, educational level, employment status, making ends meet, living area, size of SN, satisfaction with SN, emotional closeness of the closest SN member, help received, participating in social activities. Model fit (multivariate models): QoL ($R^2 = 0.43$, adjusted $R^2 = 0.43$).
